# Alprostadil for hypertensive nephropathy: A systematic review and meta-analysis of randomized controlled trials

**DOI:** 10.1371/journal.pone.0269111

**Published:** 2022-05-26

**Authors:** Hongfang Fu, Weiwei Hou, Yang Zhang, Xiaoyu Hu

**Affiliations:** 1 Infectious Disease Department, Hospital of Chengdu University of Traditional Chinese Medicine, Chengdu, Sichuan Province, People’s Republic of China; 2 School of Clinical Medicine, Chengdu University of Traditional Chinese Medicine, Chengdu, Sichuan Province, People’s Republic of China; 3 Emergency Department, Hospital of Chengdu University of Traditional Chinese Medicine, Chengdu, Sichuan Province, People’s Republic of China; The University of Mississippi Medical Center, UNITED STATES

## Abstract

We performed a meta-analysis to evaluate the efficacy of alprostadil in the treatment of hypertensive nephropathy. Seven online databases (PubMed, Embase, Cochrane Library, China National Knowledge Infrastructure [CNKI] database, Wanfang Data Knowledge Service Platform, VIP Information Resource Integration Service Platform [cqVIP], and China Biology Medicine Disc [SinoMed]) were searched from inception to January 31, 2022, and a set of clinical indicators for hypertensive nephropathy was selected. The main indicators were 24-h urinary protein, serum creatinine, endogenous serum creatinine clearance rate, blood urea nitrogen, cystatin C, and mean arterial pressure. The methodological quality of the included trials was analyzed using a risk of bias assessment according to the Cochrane Manual guidelines, and a meta-analysis was performed. A random-effects model was implemented to pool the results. A total of 20 randomized controlled trials involving 1441 patients with hypertensive nephropathy were included in this review. Our findings showed that alprostadil had a positive effect on 24-h urinary protein (mean difference [MD] = −0.79, 95% confidence interval [CI] [−1.16, −0.42], P < 0.0001), serum creatinine (MD = −13.83, 95% CI [−19.34, −8.32], P < 0.00001), endogenous serum creatinine clearance rate (MD = 6.09, 95% CI [3.59, 8.59], P < 0.00001), blood urea nitrogen (MD = −6.42, 95% CI [−8.63, −4.21], P < 0.00001), cystatin C (MD = −0.26, 95% CI [−0.34, −0.18], P < 0.00001), and mean arterial pressure levels(MD = −13.65, 95% CI [−16.08, −11.21], P < 0.00001). Compared to conventional treatment alone, alprostadil combined with conventional treatment can improve renal function in patients with hypertensive nephropathy more effectively. However, additional large-scale, multicenter, rigorously designed randomized controlled trials are needed to verify these results. This is the first meta-analysis to evaluate the efficacy of alprostadil for hypertensive nephropathy, and the results may guide clinical practice.

## Introduction

Hypertension is one of the most common cardiovascular diseases worldwide, with primary hypertension accounting for 95% of cases [1, 2]. According to the latest data, the prevalence of hypertension has nearly tripled in China over the past 30 years, with a recent survey revealing a prevalence of 27.9% in people over 18, between 2012 and 2015 [3]. Globally, more than a quarter of the world’s adult population of nearly 5 billion had hypertension in 2000, and the proportion is expected to increase to 29%, or 1.56 billion, by 2025 [4, 5]. Since hypertension progresses slowly, the disease course can be lengthy and result in serious complications in various organs. These complications can have long-term adverse effects on the health and quality of life of those affected [6]. Notably, the harm caused by secondary complications tends to be much greater than that caused by hypertension itself. One example is kidney disease, with studies showing that the incidence of chronic kidney disease in hypertensive patients is significantly higher than that in the population with normal blood pressure (16.82% and 9.06%, respectively) [7]. The literature also shows an increasing rate of hypertensive renal damage in younger patients, with an associated increase in the incidence and severity of hypertensive renal disease. It is recognized that the kidney is one of the organs most vulnerable to hypertension [8, 9].

The kidneys regulate the body’s fluid and electrolyte balance and are also important for the regulation of blood pressure [10]. Hypertensive nephropathy is a disease that occurs as a complication of essential hypertension, in which renal structure and function is damaged. Long-term high blood pressure damages the kidneys by causing renal intimal thickening, lumen stenosis, and insufficient renal blood supply, all of which can lead to ischemic nephropathy, glomerulosclerosis, renal tubular atrophy, and interstitial fibrosis in the late stages. The main pathogenesis of hypertensive nephropathy is related to hemodynamic changes, oxidative stress, inflammatory response, excessive activation of the renin-angiotensin system, and genetic and metabolic factors [11, 12]. The disease may be asymptomatic at onset, but during disease progression, manifestations such as microalbuminuria and increased blood serum creatinine (SCr) can occur. This insidious onset leads to a high rate of missed diagnoses, greatly increasing the risk of end-stage renal disease, adverse cardiovascular events, and sudden death. This poses a serious health threat to patients, has a poor prognosis, and can also become a significant economic burden [13].

The treatment of primary hypertension—blood pressure control—is an important component of the treatment of patients with hypertensive nephropathy, and the use of renin-angiotensin-aldosterone system blockers in combination with calcium channel blockers or diuretics is recommended. Among the antihypertensive drugs, angiotensin-converting enzyme inhibitors (ACEIs) and angiotensin receptor blockers (ARBs) are the first choice since they have good efficacy in hypertensive patients with target organ damage. However, renoprotective effects cannot be achieved to a satisfying extent when these drugs are used alone at the dosages recommended for blood pressure control [14]. Therefore, in addition to blood pressure control, it is both necessary and urgent to explore additional treatment options to protect kidney function.

Alprostadil, also known as prostaglandin E1, dilates the blood vessels, inhibits platelet aggregation, and improves microcirculation. Therefore, it is commonly used to treat limb ulcers caused by chronic arterial occlusion, limb pain at rest caused by impaired microvascular circulation, cardiovascular and cerebrovascular microcirculation disorders, and as anticoagulation therapy after organ transplantation [15]. Alprostadil is a promising drug for a wide range of diseases, including hepatitis, pancreatitis, diabetes-related diseases, and even erectile dysfunction in men. In recent years, the effect of alprostadil on renal function has also been investigated, with results showing that it can change renal hemodynamics by dilating glomerular afferent arterioles, and inhibiting platelet aggregation and the activity of the sympathetic renin-aldosterone system. This can delay (or even reverse, to a certain extent) the course of the disease and the extent of renal injury, and protect and stabilize residual renal function.

Many clinicians utilize alprostadil in the treatment of hypertensive nephropathy, and several randomized control trials (RCTs) have shown that compared with conventional Western medicine alone, combining alprostadil with conventional treatment (CT) is beneficial in patients with hypertensive nephropathy, improving treatment effectiveness. Even in patients with uremia [16], the combined use of alprostadil has led to an improvement in clinical symptoms, delayed the initiation of dialysis, and had no obvious side effects, making the treatment worthy of clinical attention.

Currently, there is a lack of systematic reviews and adequate evaluations of the effect of alprostadil in the treatment of hypertensive nephropathy. To fill this gap, this review used rigorous systematic evaluation and meta-analysis to evaluate the efficacy of alprostadil for the treatment of hypertensive nephropathy. The results of this study provide an evidence base for clinical treatment and further research.

## Methods

This systematic evaluation and meta-analysis was registered in the International Prospective Registry System Evaluation (PROSPERO) with registration number CRD42021286886. Further details can be found on PROSPERO. This systematic review and meta-analysis was reported according to the PRISMA statement.

### Inclusion criteria

The following inclusion criteria were used: (1) study was an RCT; (2) patients were diagnosed with hypertensive nephropathy; (3) experimental group was treated with alprostadil and the control group with a non-alprostadil alternative; (4) prognostic indicators included 24h urinary protein, SCr, endogenous serum creatinine clearance rate (Ccr), blood urea nitrogen (BUN), cystatin C, and mean arterial pressure (MAP); and (5) there were no sex, race, or nationality restrictions.

### Exclusion criteria

The exclusion criteria were as follows: (1) duplicate publications; (2) review articles, animal experiments, systematic evaluations, graduation theses, horizontal research, and conference summaries; (3) all other types of primary kidney disease, and secondary renal damage caused by secondary hypertension, diabetes, tumors, heart failure, and/or cirrhosis.

### Search strategies

Seven databases were searched from their inception to January 31, 2022: PubMed, Embase, Cochrane Library, China National Knowledge Infrastructure (CNKI) database, Wanfang Data Knowledge Service Platform, VIP Information Resource Integration Service Platform (cqVIP), and China Biology Medicine Disc (SinoMed). There was no restriction on the language of search results, and the key words used to search were: “Alprostadil” OR “Alprostadil injection” OR “prostaglandin E1” OR “PGE1,” hypertensive nephropathy” OR “hypertensive renal injury” OR “hypertensive kidney injury,” and “clinical trial” OR “RCT” OR “randomized controlled trial”.

### Data extraction

Two researchers independently screened and extracted the data based on the inclusion and exclusion criteria. A data extraction template based on the basic characteristics of the included literature was created, including country, author, publication year, number of participants, age, sex, intervention method, treatment process, and other information. Disagreements between the two authors were resolved through discussion.

### Data synthesis and analysis

All data statistics and analyses were performed by creating forest maps and performing subgroup analysis using Review Manager version 5.4 (The Nordic Cochrane Centre, Copenhagen). Relative risk was used to count data, and mean difference (MD) or standardized MD were used for measurement data. Ranges are expressed using a 95% confidence interval (CI). A random-effects model was used to calculate the effect size. Heterogeneity was tested for the included studies based on the I^2^ values and *P* values. Sensitivity analysis was conducted to validate the robustness of our results, and publication bias was assessed using funnel plot analysis.

### Quality assessment

The methodological quality of each of the included trials was assessed using the Cochrane Collaboration tool [17]. Two reviewers independently evaluated the quality of the included studies in the bias risk assessment of each trial. This was based on the following seven components: random sequence generation (selection bias), allocation concealment (selection bias), blinding of participants and personnel (performance bias), blinding of outcome evaluation (detection bias), incomplete outcome data (attrition bias), selective reporting (reporting bias), and other biases.

## Results

### Search results

**Fig 1:**
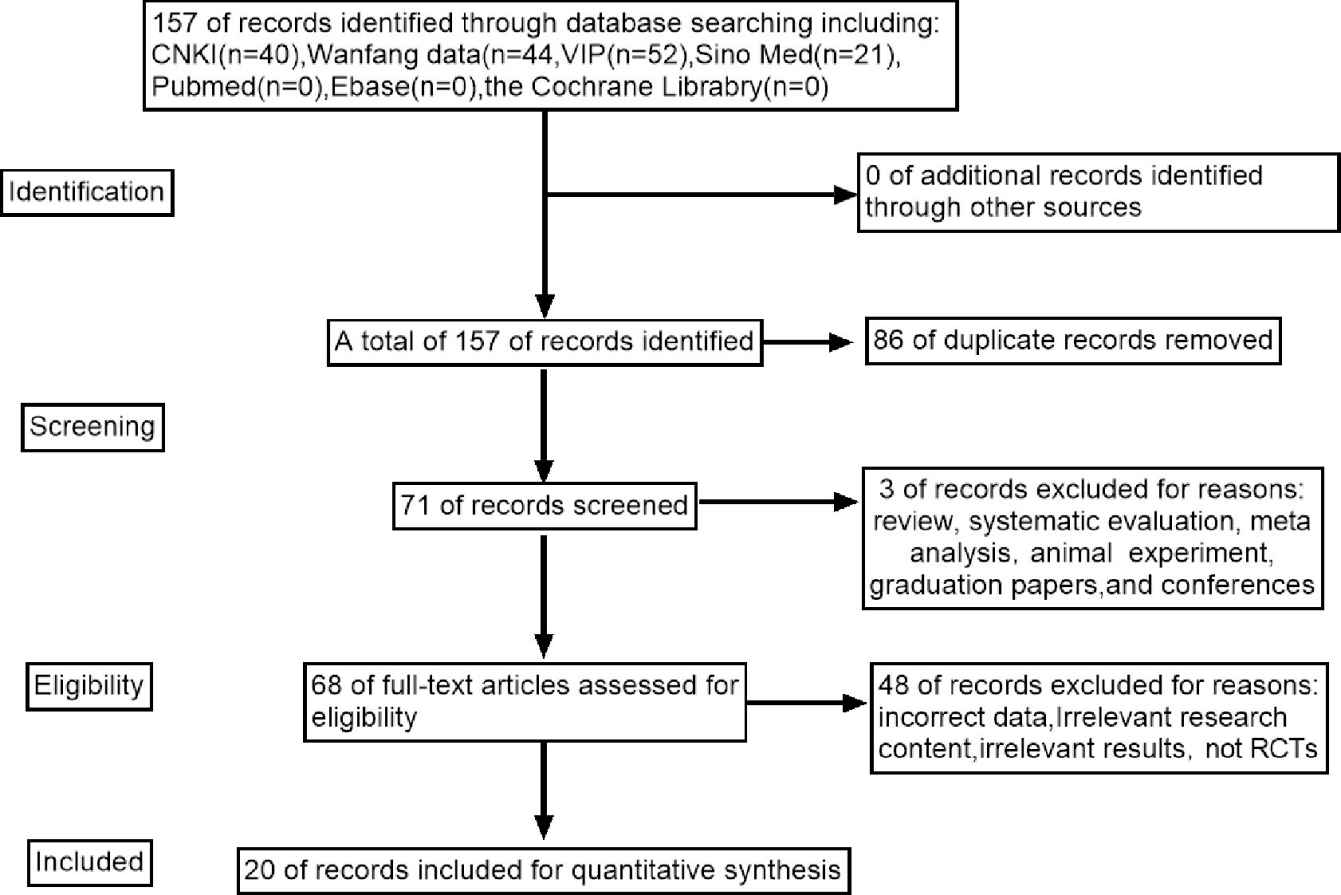
Flow diagram of the search and selection process.

The initial literature search provided 157 relevant studies. Of these, 86 duplicates were excluded and the remaining 71 were evaluated. Based on the title, abstract, and full text, an additional 51 studies were excluded due to inconsistent research content, being graduation theses, or possible data errors. Therefore, a total of 20 studies were selected for this study [18–37], including 722 participants in the experimental group and 719 in the control group, in total. The details of the selection process are illustrated in the schematic diagram in Fig 1.

### Characteristics of included studies

All 20 RCTs were conducted in China between 2003 and 2020. A total of 1441 participants aged 29–85 years, were included in the studies. Each individual study had an experimental group treated with alprostadil and a control group that did not receive alprostadil treatment. A summary of the characteristics of the studies is presented in Table 1.

**Table 1:** Characteristics of the included trials.

Author/year	Number of subjects		Number of male/female		BMI (kg/m^2^) or average weight(kg)		Average age: Mean±SD or Age range		Intervention		Treatment duration	Outcomes
	E	C	E	C	E	C	E	C	E	C		
Yang S 2016	47	47	23/24	27/20	NR		48.2±8.7	47.9±7.9	CT+Alprostadil (10 μg qd ivgtt)	CT+STS (30–50 mg qd ivgtt)	4–6 w	(4)
Zhang MB 2016	25	25	27/23		NR		63.4±6.7		CT+Alprostadil (10 μg qd ivgtt)	CT (ACEI po)	15 d	(1)(2)(5)
Xu WY 2015	35	35	38/32		NR		65.2±10.8		CT+Alprostadil (10 μg qd ivgtt)	CT (ACEI/ARB po)	15 d	(1)(2)(5)
Liu LD 2014	49	49	26/23	25/24	NR		55.8	56.9	CT+Alprostadil (10 μg qd ivgtt)	CT+DSI (60 ml qd ivgtt)	3 w	(1)(3)(4)(6)
Xu QM 2011	20	20	21/19		NR		55.7		CT+Alprostadil (10 μg qd ivgtt)	CT+DSI (60 ml qd ivgtt)	3 w	(1)(2)(3)(4)(6)
Chen QX 2012	20	20	23/17		NR		54.6±0.5		CT+Alprostadil (10 μg qd ivgtt)	CT+DSI (60 ml qd ivgtt)	3 w	(1)(2)(3)(4)(6)
Li H 2016	65	61	35/30	29/32	NR		67.5±5.2	67.8±4.9	CT+Alprostadil (10 μg qd ivgtt)	CT (ACEI po)	4 w	(1)(2)(5)
Dai G 2016	42	41	29/13	27/14	63.94±1.22	63.36±1.27	65.34±2.29	65.8±2.13	CT+Alprostadil (10 μg qd ivgtt)	CT (ACEI/ARB po)	15 d	(1)(2)(3)(5)
Tao L 2018	41	41	22/19	23/18	24.9±3.2	25.3±3.4	57.6± 15.8	58.2±16.1	Losartan +Alprostadil (10 μg qd ivgtt)	Losartan (50 mg qd po) + placebo	16 w	(2)(3)
He LH 2017	43	43	28/15	27/16	NR		62–79	61–79	ACT+Alprostadil (10 μg qd ivgtt)	ACT (10 mg qd po)	NR	(2)(4)
Liu XJ 2013	30	30	36/24	36/24	NR		NR		CT+Alprostadil (10 μg qd ivgtt)	CT	2 w	(2)
Tan ZH 2003	29	29	39/19	39/19	NR		53±7		CT+Alprostadil (100–200 μg qd ivgtt)	CT	10–14 d	(2)(4)
Xin KM 2017	45	45	25/20	24/21	NR		72.46±2.68	71.56±3.29	ACT+Alprostadil (10 μg qd ivgtt)	ACT (10 mg qd po)	2 w	(2)(4)
Jiang XL 2003	31	30	41/20		NR		69.5±8.5		CT+Alprostadil (10 μg qd iv)	CT	4 w	(2)(4)
Fu WJ 2013	35	35	21/14	20/15	NR		77±6.4	78±7.5	CT+Alprostadil (10 μg qd iv)	CT	15 d	(2)(4)
Shen RX 2018	30	30	32/28		NR		66.5±8.5		IT+Alprostadil (10 μg qd ivgtt)	IT (150 mg qd po)	2 w	(2)
Kong LS 2016	32	32	33/31		NR		45–69		IT+Alprostadil (10 μg qd ivgtt)	IT (150 mg qd po)	2 w	(2)(4)(5)
Zheng Z 2003	21	24	28/17		NR		62.37		FSRT+BHT+Alprostadil (10 μg qd ivgtt)	FSRT (5–10 mg qd po) +BHT (10 mg qd po)	2 w	(1)(2)(4)
Lu HN 2016	35	35	19/16	20/15	68.5±6.6	66.4±7.5	63.5±6.7	65.1±5.9	ACT+Alprostadil (10 μg qd ivgtt)	ACT (10 mg qd po)	2 w	(2)(4)
Sun XT 2020	47	47	26/21	25/22	NR		71.49±3.26	71.55±3.28	ACT+Alprostadil (10 μg qd ivgtt)	ACT (10 mg qd po)	NR	(2)(4)

E, experimental group; C, control group; d, days; W, weeks; qd, once daily; ivgtt, intravenous guttae; po, per os; NR, not reported.

CT, conventional treatment; STS, sodium tanshinone IIA sulfonate injection; DSI, Danshen injection; ACT, atorvastatin calcium tablets; IT, lrbesartan tablets; FSRT, felodipine sustained release tablets; BHT: benazepril hydrochloride tablets.

(1) 24-h Urinary protein; (2) SCr, (3) Ccr, (4) BUN, (5) cystatin C, (6) MAP

### Methodological quality

**Fig 2:**
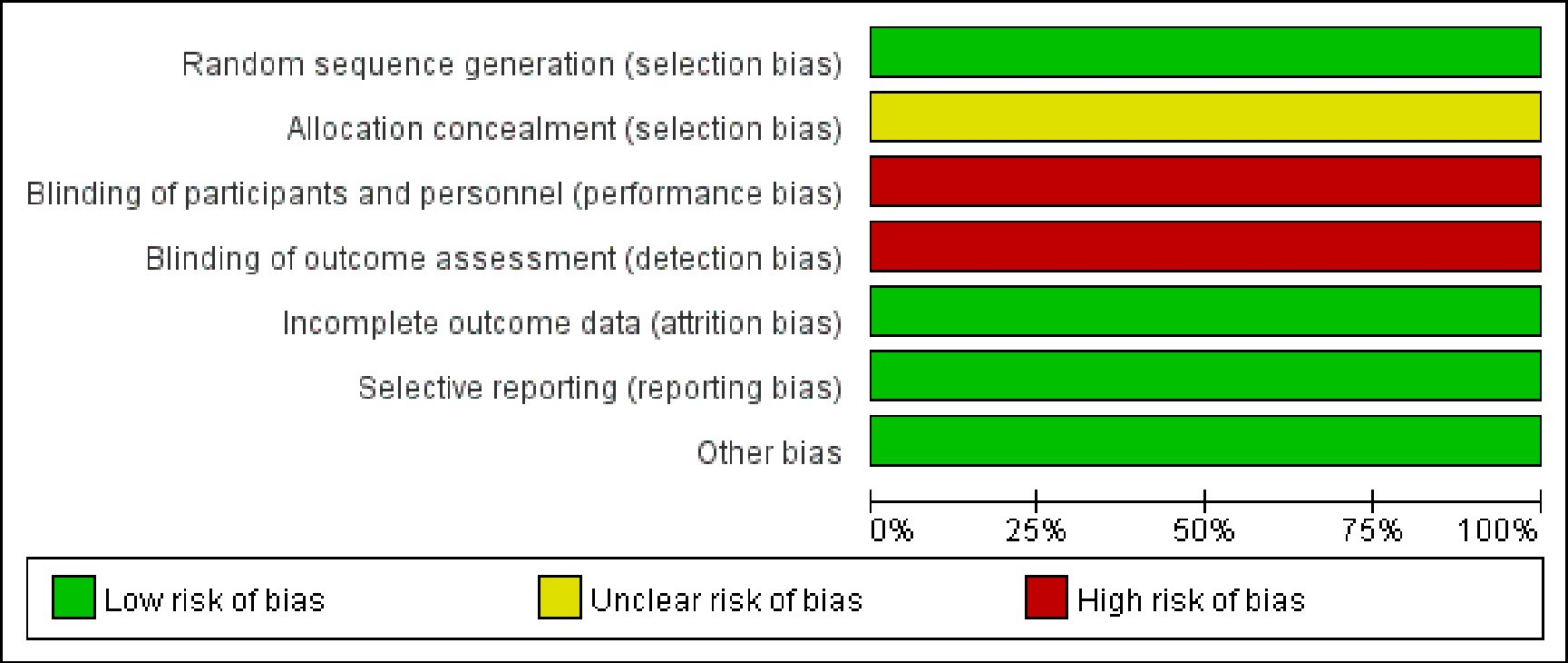
Risk of bias.

Details of the methodological quality assessment of each study are shown in Fig 2. Notably, the methodological quality assessment for each study, which was conducted using the Cochrane Collaboration tool, showed that no incomplete, selective, or other biases were found. However, there was a risk of bias in allocation concealment and blinding.

### Outcomes measured

#### 24 h urinary protein (g/24 h)

**Fig 3:**
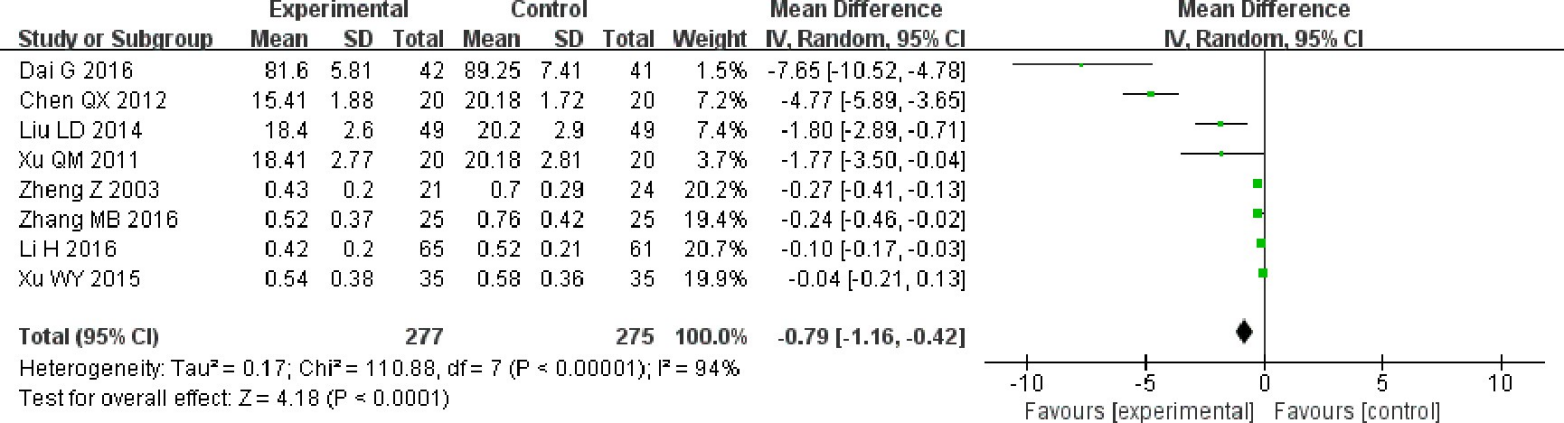
Forest plot for 24 h urinary protein.

Eight trials reported 24 h urinary protein measurements [19–25, 35]. After testing for heterogeneity (P < 0.00001, I^2^ = 94%), we adopted a random-effects model. The meta-analysis showed that 24 h urinary protein levels in the experimental group were lower than those in the control group and that the difference between the two groups was statistically significant. (MD = −0.79, 95% CI [−1.16, −0.42], P < 0.0001; Fig 3).

**Fig 4:**
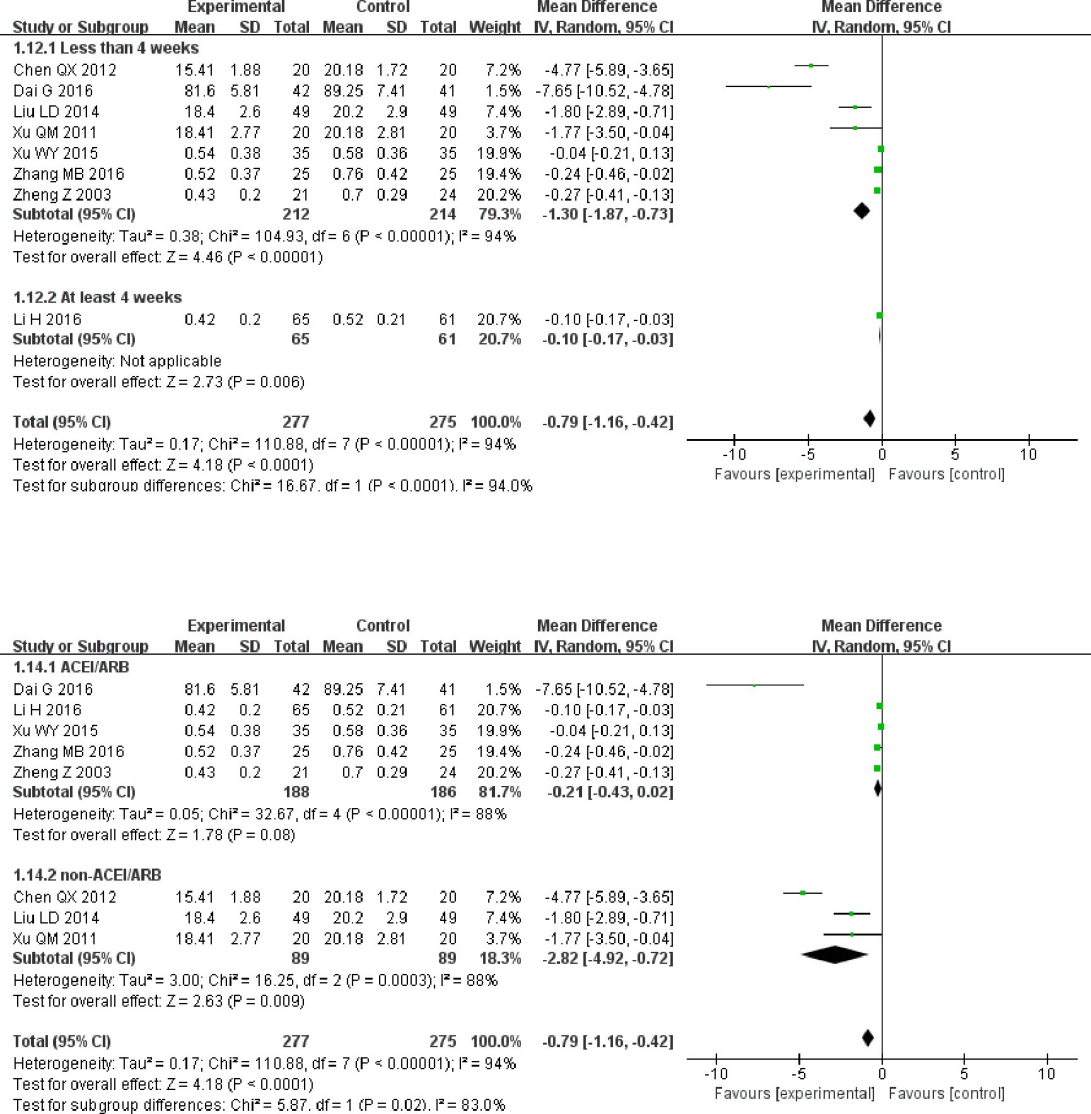
A. Forest plot of 24 h urinary protein subgroup analysis, based on treatment duration. B. Forest plot of 24 h urinary protein subgroup analysis based on treatment measures.

Due to significant heterogeneity, subgroup analysis was performed according to the different treatment durations and treatment measures of the control group. In the subgroups with treatment durations of “less than 4 weeks” and “at least 4 weeks,” the effect of alprostadil on reducing 24 h urinary protein in the experimental group was significantly better than that in the control group (MD = −1.30, 95% CI [−1.87, −0.73]), (MD = −0.10, 95% CI [−0.17, −0.03]; Fig 4A).

Based on the subgroup analysis of whether ACEIs or ARBs were utilized, the results suggest that if not treated with ACEI/ARB, alprostadil was more effective in reducing 24 h urinary protein in the experimental group (MD = −2.82, 95% CI [−4.92, −0.72]; Fig 4B).

Therefore, we can conclude that it is likely that the heterogeneity of the 24 h urinary protein results is related to treatment duration and the use of ACEIs/ARBs in the control group.

Sensitivity analysis was performed on the 24 h urinary protein data by excluding each trial individually and re-analyzing the remaining trials to ascertain whether there was a significant change in the results. The sensitivity analysis suggested that the results were stable.

#### SCr (μmoL/L)`

**Fig 5:**
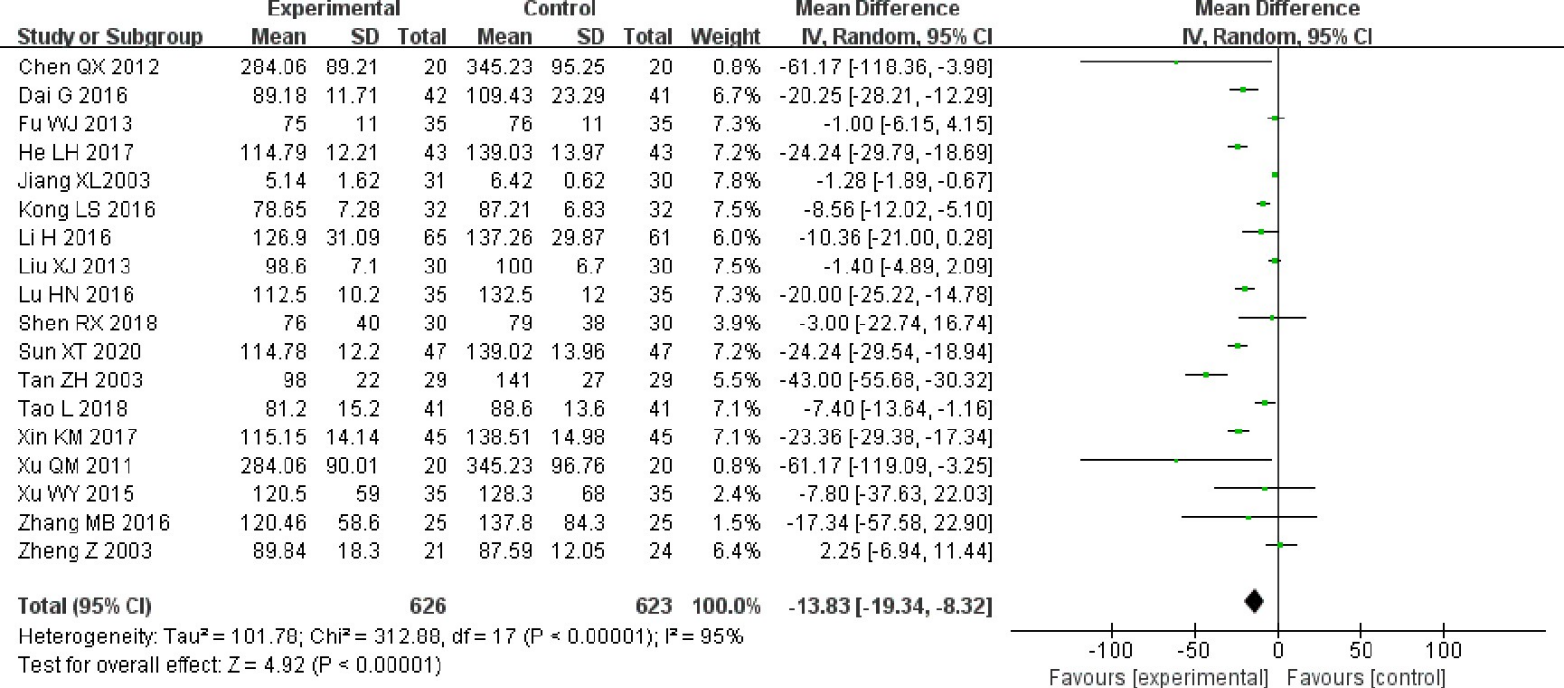
Forest plot for SCr.

All 18 clinical trials reported on SCr [19, 20, 22–37]. After testing for heterogeneity (P < 0.00001, I^2^ = 95%), the random-effects model was used to estimate the size of the combined effect. The results showed that, compared with the control group, SCr improved significantly more in the experimental group (MD = −13.83, 95% CI [−19.34, −8.32], P < 0.00001; Fig 5).

**Fig 6:**
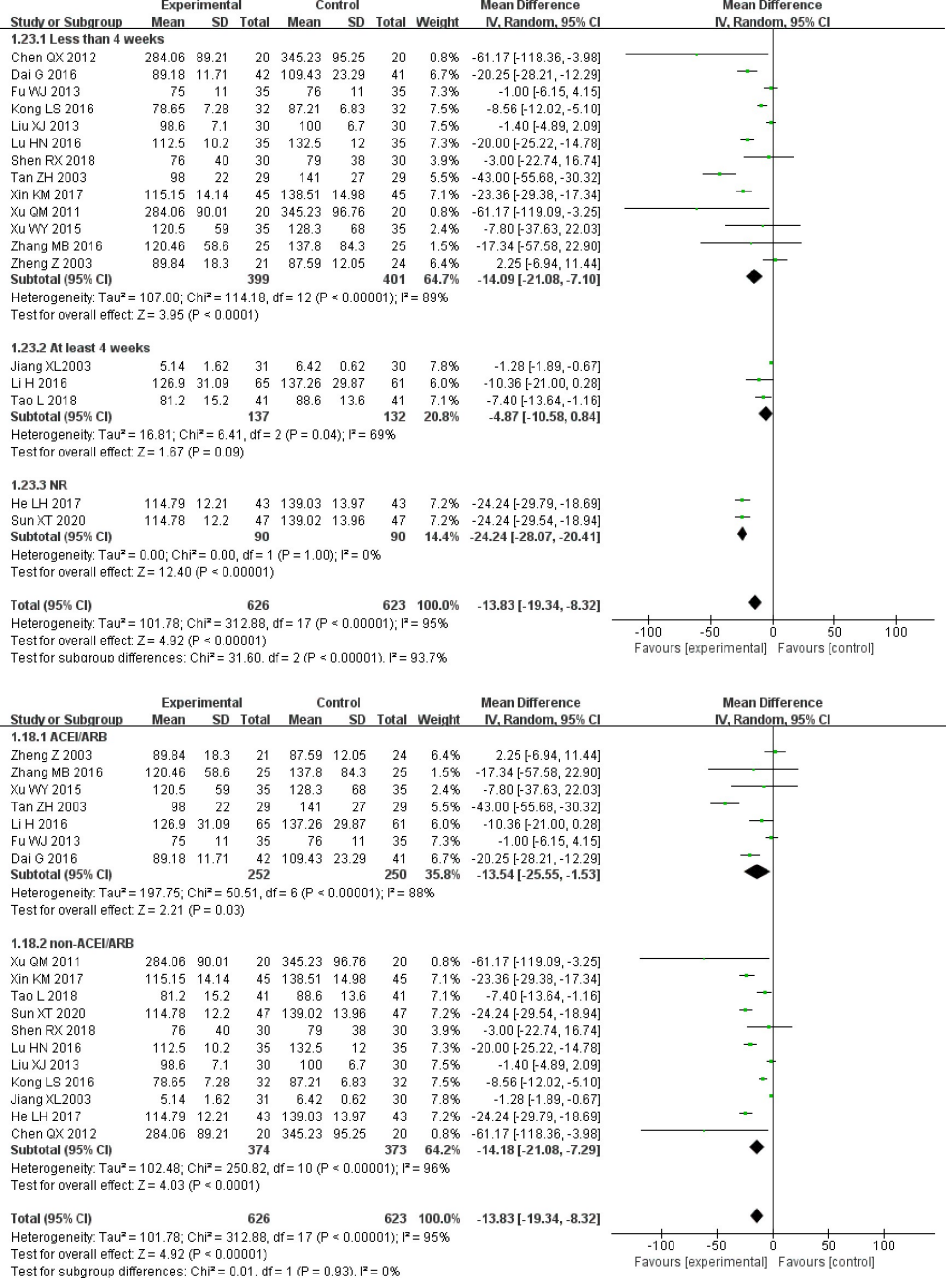
A. Forest plot of SCr subgroup analysis based on treatment duration. B. Forest plot of SCr subgroup analysis based on treatment measures.

As heterogeneity was statistically significant, a subgroup analysis was conducted based on treatment duration. In the “less than 4 weeks” and “NR” subgroups, combined treatment was associated with a significantly larger improvement in SCr compared to control group treatment alone (MD = −14.09, 95% CI [−21.08, −7.10]), (MD = −24.24, 95% CI [−28.07, −20.41]). In the “at least 4 weeks” subgroup, the combination therapy appeared to reduce the improvement in SCr; however, the difference between the two subgroups was not statistically significant (MD = −4.87, 95% CI [−10.58, 0.84]; Fig 6A).

When basing the subgroup analysis on whether the control group utilized ACEI/ARB, we found that in both the “ACEI/ARB” and “non-ACEI/ARB” subgroups, the decrease in SCr in the experimental group was significantly higher than that in the control group (MD = −13.54, 95% CI [−25.55, −1.53]), (MD = −14.18, 95% CI [−21.08, −7.29]; Fig 6B).

According to the results of the subgroup analysis above, we can conclude that the heterogeneity in SCr data is related to treatment duration and is not likely to be related to ACEIs/ARBs use in the control group.

A sensitivity analysis of SCr validated the robustness of the results.

**Fig 7:**
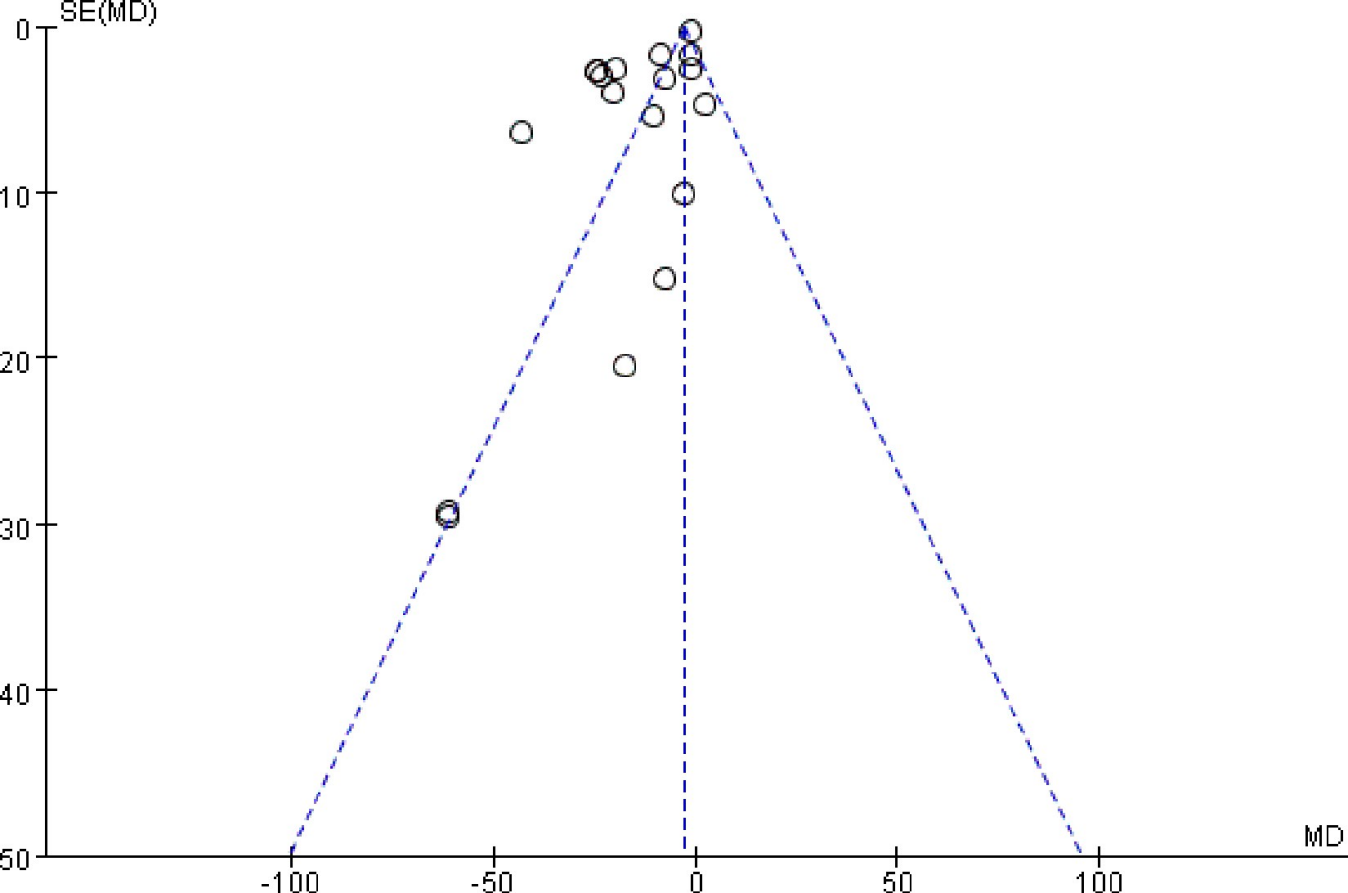
Funnel plot for the publication bias of SCr.

The funnel plot was asymmetric, which suggested that there was likely a publication bias between the SCr results (Fig 7).

#### Ccr (ml/min)

**Fig 8:**
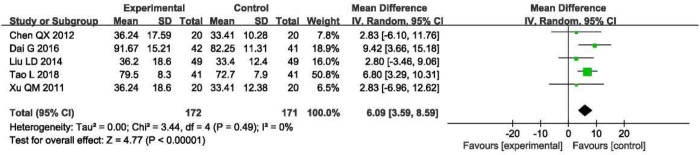
Forest plot of Ccr.

Five studies reported Ccr data [21–23, 25, 26]. As there was no heterogeneity among the studies (P = 0.49, I^2^ = 0%), the random-effects model was still used, and the results showed that the combination therapy was more effective than that in the control group in improving Ccr (MD = 6.09, 95% CI [3.59, 8.59], P < 0.00001; Fig 8).

The sensitivity analysis suggested that the results were stable. Because the number of RCTs included in the Ccr counts was less than 10, there was no funnel chart analysis for this part of the data.

#### BUN (mmol/L)

**Fig 9:**
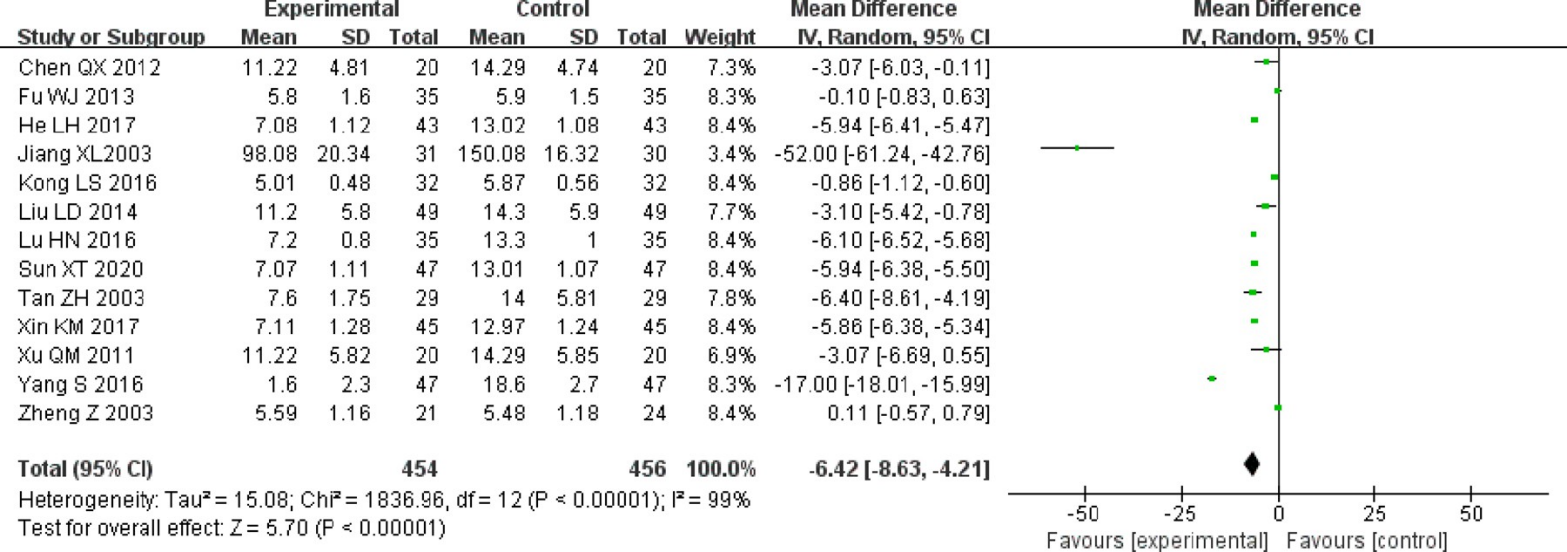
Forest plot of BUN.

BUN levels were reported in 13 of the included trials [18, 21–23, 27, 29–32, 34–37]. After testing for heterogeneity (P < 0.00001, I^2^ = 99%), the random-effects model was used to calculate the comprehensive effect size. The results suggested that the BUN levels of the experimental group were lower than those of the control group; and the difference between the two groups was statistically significant (MD = −6.42, 95% CI [−8.63, −4.21], P < 0.00001; Fig 9)

**Fig 10:**
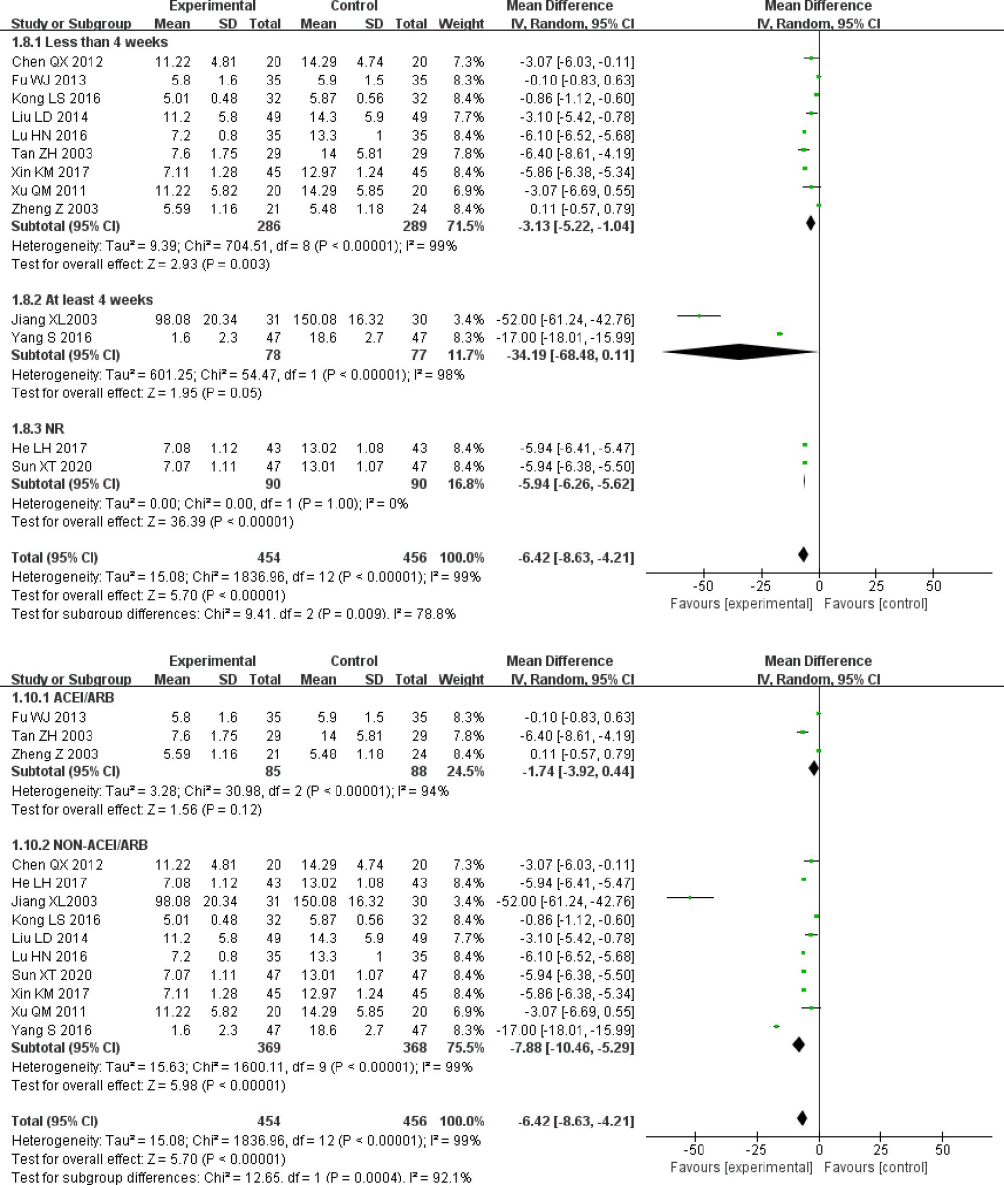
A. Forest plot of BUN subgroup analysis based on treatment duration. B. Forest plot of BUN subgroup analysis based on treatment measures.

Due to high heterogeneity, a subgroup analysis was conducted based on the course of treatment and treatment measures. In the “less than 4 weeks” and “NR” subgroups, those undergoing combined treatment showed significantly better improvement in BUN than those on control group treatment alone (MD = −3.13, 95% CI [−5.22, −1.04]), (MD = −5.94, 95% CI [−6.26, −5.62]). In the “at least 4 weeks” subgroup, there was no statistically significant difference between the two groups (MD = −34.19, 95% CI [−68.48, 0.11]; Fig 10A].

Subgroup analysis based on treatment measures showed that with respect to “ACEI/ARB,” use there was no significant difference between the experimental group and the control group (MD = −1.74, 95% CI [−3.92, 0.44]). However, in the case of "non-ACEI/ARB,” the use of alprostadil improved the BUN of patients significantly (MD = −7.88, 95% CI [−10.46, −5.29]; Fig 10B).

In conclusion, the heterogeneity of BUN levels may be related to the course of treatment and treatment measures.

Sensitivity analysis showed that the results were stable.

**Fig 11:**
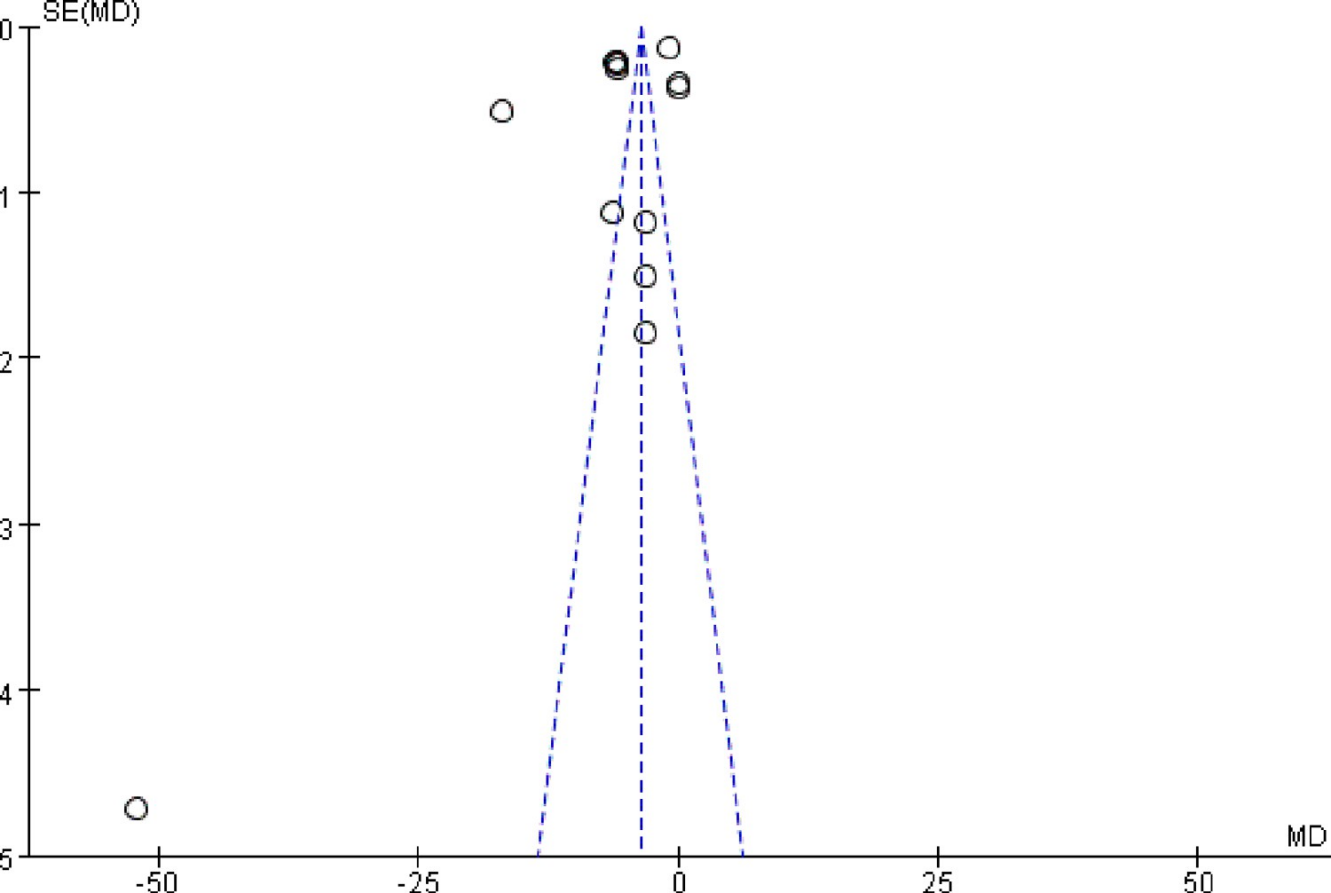
Funnel plot for the publication bias of BUN.

The funnel plot was symmetrical, suggesting that there was no publication bias (Fig 11).

#### Cystatin C (mg/L)

**Fig 12:**
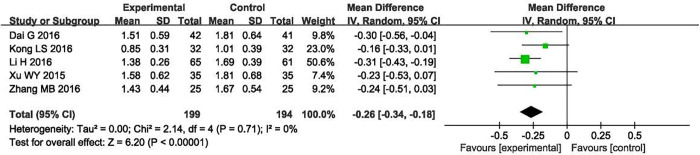
Forest plot of cystatin C.

Cystatin C results were recorded in five of the included studies [19, 20, 24, 25, 34]. Our analysis found no heterogeneity (P = 0.71, I^2^ = 0%); the random-effects model was still used to summarize the data. The results showed that the experimental group was superior to the control group in the reduction of cystatin C and that the difference was statistically significant (MD = −0.26, 95% CI [−0.34, −0.18], P < 0.00001; Fig 12).

Sensitivity analysis showed that the results were stable, and a test for publication bias was not performed, as the total number of studies included in our analysis was less than 10.

#### MAP (mm Hg)

**Fig 13:**
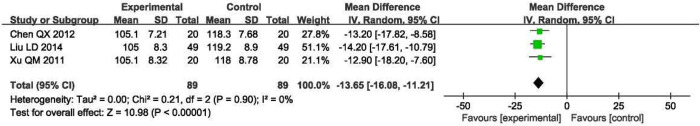
Forest plot of MAP.

Only three clinical trials included MAP data [21–23]. There was no heterogeneity (p = 0.90, I^2^ = 0%); the random-effects model was still adopted. The results showed that, compared with the control group, the MAP level of patients treated with alprostadil decreased significantly (MD = −13.65, 95% CI [−16.08, −11.21], P < 0.00001; Fig 13). The sensitivity analysis suggested that the results were stable. Publication bias tests were not performed.

## Adverse events

Common clinical adverse reactions to alprostadil included pain in the blood vessels at the injection site, phlebitis, flushing of the face, dizziness, palpitations, nausea, and pruritus. Of the 20 clinical trials, five trials (429 patients) reported no adverse reactions, and seven trials (502 patients) clearly demonstrated high safety and no adverse reactions. In the other eight studies (510 patients), 18 patients in the experimental group experienced vascular pain at the injection site, three experienced dizziness and headache, and two experienced nausea; while in the control group there was one case each of facial flushing, headache, vomiting, diarrhea, and palpitations. These adverse reactions were well tolerated by the patients, and no serious adverse reactions were observed. The symptoms disappeared after slowing down the administration rate or relieved spontaneously without special treatment; therefore, no patients were terminated or withdrawn from the trials.

## Discussion

Hypertension has a long disease course with slow progression. Hypertension generates a serious economic burden on society owing to its high incidence and numerous complications, including kidney damage caused by intra-glomerular hypertension. Hypertensive nephropathy has a complex pathogenesis and is often undiagnosed in its early stages since it is often asymptomatic at onset [38]. However, early and aggressive treatment is critical to minimize further kidney damage in these patients. In the current literature, the prognosis of patients with hypertensive nephropathy is clearly related to clinical indicators such as 24 h urinary protein, SCr, Ccr, cystatin C, and BUN [39, 40].

Systematic reviews and meta-analyses are vital to top level clinical research. In this study, we conducted a meta-analysis based on 20 RCTs to determine the efficacy of alprostadil in patients with hypertensive nephropathy by using six indicators of hypertensive nephropathy: 24-hour urinary protein, SCr, Ccr, BUN, cystatin C and MAP. We found that alprostadil combined therapy could improve 24 h urinary protein, SCr, Ccr, BUN, cystatin C, and MAP, as well as improving renal function in patients. There was no significant heterogeneity in Ccr, cystatin C, or MAP results. However, significant heterogeneity was detected in the 24 h urinary protein, SCr, and BUN levels, and subgroup analysis showed that this was related to treatment duration and whether the control group used ACEIs/ARBs.

The sensitivity analysis of all six indicators showed that the results were stable, although the asymmetric funnel plot of SCr suggested the possibility of publication bias. The symmetry of the BUN funnel plot suggests that there was no publication bias. The remaining indicators were not evaluated for publication bias because there were fewer than 10 papers in the literature.

The adverse reactions to alprostadil were mainly pain and flushing at the injection site, but the symptoms were relieved after slow intravenous drip. No serious adverse reactions were observed, indicating the high safety of alprostadil.

Fortunately, alprostadil has been used with lipid microsphere carrier preparations, which can effectively reduce adverse reactions, such as vascular pain, and the unstable chemical properties of prostaglandin E1. The modified alprostadil is more effective, targeted, and persistent, and can directly affect glomerular afferent arterioles and increase renal blood flow and the glomerular filtration rate. Therefore, it has high application value and is worthy of clinical attention.

## Limitations

Although we consistently and thoroughly interpreted and reported the results in this study, its limitations must be acknowledged. First, all the included studies were published in Chinese and all participants were Chinese; therefore, the applicability to patients in other regions is limited and requires further study. Second, the methodological quality of the included studies was generally low. All included trials were missing important methodological details, such as allocation concealment, blinding of participants and personnel, and blinding of outcome evaluations—leading to unreliable results. To verify our findings, further large sample, multicenter, high-quality RCTs are needed. Finally, none of the clinical trials included in our analysis were publicly registered; therefore, we are unable to rule out the possibility of publication bias. Despite these limitations, our study is the first systematic evaluation of the efficacy of alprostadil in the treatment of hypertensive nephropathy and may be of use to clinicians.

## Conclusions

This systematic review and meta-analysis assessed the effects of alprostadil on hypertensive nephropathy by comprehensively analyzing the results of a selection of RCTs. According to the results of this meta-analysis, alprostadil combined with CT had a greater beneficial effect than CT alone on 24 h urinary protein, SCr, Ccr, cystatin C, BUN, and MAP—all of which are important markers of kidney function. Although alprostadil was shown to be effective in the treatment of hypertensive nephropathy in all 20 studies, this needs to be verified in large-scale, multicenter, randomized, double-blind clinical trials. Simultaneously, additional studies are recommended to further evaluate the safety of this drug in patients with hypertensive nephropathy.

## Supporting information

S1 Checklist PRISMA 2020 checklist. (DOCX)
